# Reviewed article: Cardins KKB, Silva IS, Soares A, Carvalho LMA, Graça JMB, Freitas CHSM. Factors associated with post-COVID-19 health conditions at a rehabilitation center: a cross-sectional study, Campina Grande, Paraíba, Brazil, 2024. Epidemiol Serv Saude. 2026:35;e20250305.

**DOI:** 10.1590/S2237-96222026v35e20250305.b

**Published:** 2026-04-20

**Authors:** Mariana Del Grossi Moura

**Affiliations:** 1 Universidade de Sorocaba, Sorocaba, SP, Brazil Universidade de Sorocaba Sorocaba SP Brazil

## First round comments

Agradecemos a submissão do manuscrito RESS-2025-0503 intitulado “Condições de saúde pós-infecção por covid-19: estudo transversal em um centro de reabilitação em Campina Grande, Paraíba, 2024” à Revista Epidemiologia e Serviços de Saúde: revista do SUS (RESS).

Após a revisão do seu artigo, identificamos a necessidade de uma grande revisão (Major Revision) para que o manuscrito esteja de acordo com os padrões editoriais da revista e possa ser viabilizado para publicação.

Além dos pontos detalhados pelos pareceristas, reforço os seguintes aspectos fundamentais que devem ser ajustados:

Título: reflita se o título não deve representar com maior precisão os objetivos e resultados do estudo, especialmente no que se refere à análise dos fatores associados ao desfecho do tratamento.

Resumo: deve ser estruturado de forma clara, contendo as seções: introdução, objetivo, método, resultados e conclusão,

Resumo (resultados): iniciar esta seção informando o total de participantes incluídos no estudo, de forma clara e objetiva. Além disso, é importante inserir os dados numéricos das análises realizadas, como proporções e, quando aplicável, medidas de associação e seus respectivos valores (ex.: razão de chances, razão de prevalência, IC95%), para sustentar as afirmações sobre fatores associados ao desfecho do tratamento.

Resumo (conclusão): revisar de modo que esteja alinhada ao objetivo do estudo e estritamente fundamentada nos principais achados apresentados. A versão atual apresenta caráter genérico, com menção ampla à “complexidade” da reabilitação e à necessidade de abordagem multidisciplinar, sem retomar os fatores estatisticamente associados ao desfecho do tratamento, que constituem o foco central do estudo. Deve restringir-se aos resultados efetivamente encontrados, evitando extrapolações.

Introdução: não está plenamente adequada ao objetivo proposto, especialmente no que se refere à justificativa para a realização do estudo A seção deve explicitar de forma mais clara por que é necessário conhecer o perfil das pessoas com condições pós-covid-19 atendidas em centros de reabilitação e qual a relevância prática ou científica de identificar os fatores associados ao desfecho do tratamento.

Método: precisa de melhorias substanciais

- Ajustar o delineamento para “estudo transversal”, evitando expressões redundantes como “estudo quantitativo, descritivo do tipo transversal”, e padronizar esse termo no resumo e na seção de métodos.

- Revisar com base no checklist STROBE, desmembrando e detalhando os seguintes itens: delineamento, contexto (local e período), participantes (critérios de inclusão/exclusão), variáveis (listadas conforme categorias apresentadas nos resultados), fontes de dados e mensuração, possíveis vieses, tamanho do estudo e métodos estatísticos

- Remover informações éticas da seção de métodos, pois essas já constam na folha de rosto.

- Faltam informações mais precisas sobre o funcionamento do programa, o tipo de unidade (pública/privada), abrangência e critérios de acesso.

- Não está claro quantos prontuários foram incluídos inicialmente, se houve perdas ou exclusões por outros motivos (ex: dados incompletos).

- A fonte populacional está limitada ao programa, mas seria importante dizer como os pacientes eram encaminhados ou selecionados para esse serviço.

- É necessário listar explicitamente todas as variáveis analisadas, com suas categorias, conforme apresentadas nas tabelas.

- Não há qualquer menção a potenciais vieses, como viés de seleção, viés de informação (dados incompletos dos prontuários), ou estratégias adotadas para minimizar esses riscos.

- Informar o número total de prontuários incluídos, e o cálculo amostral ou uso de amostra por conveniência (o que parece ser o caso).

- Para análise estatística, faltam as medidas de associação utilizadas (ex.: RP, OR, IC95%). Elas devem ser descritas em métodos e não apenas nos resultados.

Resultados:

- Expressões como “foram realizadas as discussões com base nos achados” devem ser removidas dos resultados - pertencem à seção de discussão.

- Embora os p-valores estejam indicados, não foram apresentadas as medidas de associação (ex.: razão de chances ou de prevalência, IC95%), que são fundamentais para interpretar a magnitude das associações. A ausência dessas medidas torna a análise superficial e dificulta a avaliação do impacto das variáveis sobre o desfechos.

- Muitos dados descritos no texto já estão (presumivelmente) nas tabelas. O ideal é resumir os achados principais no texto e referenciar as tabelas, sem duplicar. Exemplo: "As principais comorbidades foram sinusite (44,1%), hipertensão arterial sistêmica (40,9%) e diabetes (23,3%) (Tabela 1)."

- O desfecho está definido como “conclusão por alta ou abandono”, mas não está claro como essas categorias foram operacionalizadas. Isso deveria ter sido esclarecido nos métodos.

Tabelas: revisar a apresentação das tabelas para garantir clareza, padronização e aderência aos critérios editoriais da RESS. Necessária a inclusão de medidas de associação com seus respectivos intervalos de confiança, principalmente nas tabelas que apresentam testes de associação. Considerar juntar comorbidades pouco frequentes em “outras” ou justificar sua análise separada. Revisar as notas de rodapé para indicar claramente o número de observações válidas, e reorganizar as variáveis por blocos temáticos

Títulos de tabelas: revisar os títulos das tabelas para garantir que sejam autossuficientes, conforme as diretrizes editoriais. Os títulos devem informar claramente o conteúdo apresentado, a população analisada, o local, o período do estudo e o número de participantes considerados em cada tabela. Por exemplo, títulos genéricos como “Tabela 2. Internação e características da assistência” não explicam o que está sendo comparado ou medido. Sugere-se ainda explicitar as variáveis analisadas, o desfecho de interesse, o tipo de análise apresentada (ex.: associação bivariada) e eventuais subconjuntos da amostra utilizados (quando n ≠ total do estudo).

Discussão: A discussão mistura resultados, revisão de literatura e interpretação de forma difusa, dificultando a fluidez e a compreensão. Falta um encadeamento lógico entre os parágrafos. Reorganizar o texto para garantir maior clareza, evitar repetições com os resultados e articular melhor os achados com a literatura científica de forma crítica e objetiva. É importante explorar com maior profundidade o significado dos achados estatisticamente significativos, refletir sobre suas implicações práticas e reforçar os limites metodológicos do estudo. A conclusão deve retomar uma síntese dos principais achados e suas contribuições no contexto do SUS.

Solicitamos que todos os comentários da revisão por pares sejam respondidos pontualmente por meio de uma carta resposta detalhada, explicando as modificações realizadas ou justificando, quando necessário, a manutenção do texto original. A nova versão do manuscrito será reconsiderada após essa reformulação e poderá passar por nova rodada de avaliação. Destacamos que esta decisão não garante a aceitação final do manuscrito.

Recommendation: Major revision

## Second round comments

Agradecemos a submissão do manuscrito RESS-2025-0503.R1 intitulado “Condições de saúde pós-COVID-19: fatores associados ao estágio do tratamento em centro de reabilitação, Campina Grande, Paraíba, 2024” à Revista Epidemiologia e Serviços de Saúde: revista do SUS (RESS).

Após a revisão de seu artigo, identificamos melhorias significativas porém alguns aspectos ainda requerem atenção para que o manuscrito esteja plenamente em conformidade com os padrões científicos e editoriais da RESS e possa ser viabilizado para publicação.

- Título: deve apresentar tema principal, delineamento, local e ano(s) da pesquisa, conforme o guia de redação. Sugestão de reescrita: “Fatores associados às condições de saúde pós-COVID-19: estudo transversal em um centro de reabilitação em Campina Grande, Paraíba, 2024.”

- Resumo (objetivo): padronizar o texto em todas as seções do manuscrito, esta faltando o artigo “o”. Correção sugerida - Objetivo: Caracterizar o perfil das pessoas com condições pós-COVID-19 e os fatores associados ao desfecho do tratamento em um centro de reabilitação no município de Campina Grande, Paraíba.

- Método: Nos métodos, os desfechos do estudo foram corretamente definidos como “alta” e “abandono”, o que é coerente com o objetivo de identificar fatores associados ao desfecho do tratamento. No entanto, na subseção “Análise estatística” os desfechos de interesse são apresnetados como “sequelas e reinternação”.

- No resumo é importante explicitar de forma clara qual foi o desfecho analisado no estudo. Recomenda-se inserir na parte de métodos, uma frase breve que defina claramente o desfecho analisado, por exemplo: “O desfecho de interesse foi o resultado do tratamento, categorizado como alta ou abandono.” Nos resultados do resumo, é importante indicar a distribuição geral das duas categorias do desfecho, o que torna o texto mais completo e transparente. Sugere-se acrescentar: “Dos 398 pacientes analisados, 282 (78,1%) receberam alta e 79 (21,9%) abandonaram o tratamento.”

- Resultados (texto): No corpo do texto, o desfecho já está parcialmente mencionado na frase “78,1% concluíram o tratamento (Tabela 2)”. Entretanto, a redação atual obriga o leitor a consultar a tabela para saber que “concluiram o tratamento” corresponde à categoria “alta” e que existe outra categoria (“abandono”) representando 21,9% dos casos. Reescreva essa parte do parágrafo para incluir as duas categorias de forma explícita, substituindo a frase atual por exemplo por: “Dos 398 prontuários analisados, 282 (78,1%) pacientes receberam alta e 79 (21,9%) abandonaram o tratamento.”

- Na Tabela 4, todos os pacientes com bronquiectasia estão na categoria “abandono”, mas o texto afirma: “...apresentou uma associação significativa com a conclusão do tratamento (RP=2,05; IC95% 1,34; 3,12; p=0,004), uma vez que todos os pacientes abandonaram o tratamento.” Isso parece contraditório: o valor de RP>1 indica associação com abandono, não com conclusão. Escreva com maior clareza visto que o correto seria algo como “A presença de bronquiectasia esteve associada a maior prevalência de abandono do tratamento, enquanto sua ausência se relacionou à conclusão do acompanhamento.”

- Discussão: Após os ajustes realizados na seção de Resultados, recomenda-se reorganizar a Discussão para assegurar coerência com os achados atualizados. A interpretação deve refletir corretamente os resultados corrigidos. Sugere-se ainda manter a discussão concisa e centrada na interpretação crítica dos achados, evitando repetições dos resultados.

Os comentários da revisão devem ser respondidos pontualmente por meio de uma carta resposta detalhada, explicando as modificações realizadas ou justificando, quando necessário, a manutenção do texto original.

Recommendation: Minor revision

